# Research on the influence and mechanism of human–vehicle moral matching on trust in autonomous vehicles

**DOI:** 10.3389/fpsyg.2023.1071872

**Published:** 2023-05-30

**Authors:** Na Chen, Yao Zu, Jing Song

**Affiliations:** ^1^College of Economics and Management, Beijing University of Chemical Technology, Beijing, China; ^2^Management College, Beijing Union University, Beijing, China

**Keywords:** deontology and utilitarian morality, perceived value, perceived risk, selfish preference, autonomous vehicles, trust

## Abstract

**Introduction:**

Autonomous vehicles can have social attributes and make ethical decisions during driving. In this study, we investigated the impact of human-vehicle moral matching on trust in autonomous vehicles and its mechanism.

**Methods:**

A 2*2 experiment involving 200 participants was conducted.

**Results:**

The results of the data analysis show that utilitarian moral individuals have greater trust than deontological moral individuals. Perceived value and perceived risk play a double-edged role in people’s trust in autonomous vehicles. People’s moral type has a positive impact on trust through perceived value and a negative impact through perceived risk. Vehicle moral type moderates the impact of human moral type on trust through perceived value and perceived risk.

**Discussion:**

The conclusion shows that heterogeneous moral matching (people are utilitarian, vehicles are deontology) has a more positive effect on trust than homogenous moral matching (both people and vehicles are deontology or utilitarian), which is consistent with the assumption of selfish preferences of individuals. The results of this study provide theoretical expansion for the fields related to human-vehicle interaction and AI social attributes and provide exploratory suggestions for the functional design of autonomous vehicles.

## 1. Introduction

With the rapid development of artificial intelligence (AI) technology, autonomous vehicles are also moving from a lower level such as assisted driving to a higher level. High-grade autonomous vehicles have the abilities of reasoning, planning, autonomous learning and behavioral decision-making. They can complete the handover of driving tasks to the driver at the right time, which means that people have changed from being machine manipulators to being partners with agents that share their decisions with them. This change shift puts forward a new challenge to human–vehicle interaction designs (Maurer et al., 2016). Meanwhile, AI machines with anthropomorphic features and social attributes, such as autonomous vehicles, can have moral character information. They can comply with social and moral rules and interact with human beings to a certain extent (Moor, 2006). Improving people’s trust in autonomous vehicles can improve the efficiency and effectiveness of human–vehicle sharing decision-making to better achieve the purpose of efficient and safe driving (Waldrop, 2015), which is also an important guarantee for improving the public’s acceptance of autonomous vehicles (Adnan et al., 2018).

Trust is the foundation of relationships that involve transactions or exchanges and is usually built in a gradual manner, generating change as conditions change (Zucker, 1986). Trust is dynamic and requires constant two-way interaction (Cheng et al., 2021). Trust in interpersonal relationships is mainly influenced by human and social environments (Jing et al., 2020). Frequent interaction between the two parties and an increase in interaction time can improve interpersonal trust, and the social environment of interaction also has an impact on trust (Heimer, 2001; Smets et al., 2013). While in the human-machine trust relationship, in addition to people and the environment, the transaction or exchange process also involves artificial intelligence/machines. The continuous two-way interaction between humans and machines/computers is accompanied by a change in human trust in related technologies. Trust is the most prominent feature of an ideal relationship (Rempel et al., 1985), and a lack of trust in human-vehicle sharing decisions may cause serious social order problems. When the moral behavior of an AI machine does not conform to human expectations, it is necessary to explain its decision accordingly to gain human trust. The social dilemma of self-driving vehicles, that is, the conflict between individual and collective interests in the context of autonomous vehicles technology, illustrates this challenge (Bonnefon et al., 2016). Furthermore, the trolley problem in the context of autonomous vehicles, the combination of different choices to save and sacrifice lives, is a moral embodiment of traffic behavior.

Morality is an important social rule that maintains the internal balance of society, plans the internal order of society and promotes social harmony (Curry, 2016). The fundamental purpose of morality is to coordinate and deal with human interpersonal relationships. In human society, morality provides powerful support for the judgment of interpersonal relations (Brambilla and Leach, 2014). Compared with nonmoral information, moral information has a more direct impact on the impression formation of others (Van Bavel et al., 2012). However, the moral rules and attributes of human society may not be fully applicable in the field of artificial intelligence, where people apply different ethics to other people than to AI. People expect AI machines to make utilitarian choices based on the premise that the driver’s interests are ensured to the greatest extent possible. Instead, people prefer to take autonomous vehicles that protect passengers at all costs (Bonnefon et al., 2016; Shariff et al., 2017).

The discussion on autonomous vehicles and ethical issues is attracting more and more scholars’ attention. The moral dilemma is one of the key issues discussed by scholars. A “moral dilemma” is when an individual is caught between two clearly conflicting moral imperatives, and if one obeys one violates the other, the choice he or she makes may conflict with his or her own values and morals (Greene et al., 2001). The trolley problem is a typical example of the “moral dilemma” (Foot, 1967). This scenario refers to the individual faced with the choice of whether to operate the train track switching lever, without which the incoming train will travel along the established track—which will result in the death of five innocent people tied to the established track. If the lever is manipulated, the train will veer off and head to another track—which will lead to the death of an innocent person tied to the other track. In the trolley problem, the different decisions of individuals (operate the lever or not) represent different moral preferences, such as utilitarian morality and deontological moral. From a utilitarian moral point of view, individuals should operate the lever to make the train go on another track, which can save the lives of most people. From the point of view of deontological moral, the individual should not operate, because everyone has equal rights, and the rights of five people on the established track and one person on the other track are also equal. This dilemma of opposing moral choices is an important foundation for research on the ethics of autonomous vehicles.

Morality affects trust, and the degree to which both parties agree with each other’s morality in interpersonal relationships is a decisive feature of high-trust relationships (Lewicki et al., 1998). An individual’s decisions reflect his or her morals (Klöckner et al., 2013; Chen and Tung, 2014). When a person makes choices as a single individual, his or her behavioral decision is usually simple without considering the allocation of public resources and the impact on others. However, when people are in social environments, their behavioral decisions not only affect them as individuals but also affect other people, including the collective in the social environment. Some studies have pointed out that in addition to morality itself, the influence of morality on trust in interpersonal relationships is influenced by many other factors, including cultural background, moral context, social environment (Hogler et al., 2013). However, in human–computer relationships, this kind of influence is affected by the moral situation, machine appearance, and degree of machine personification (Komatsu, 2016; Malle et al., 2016; Awad et al., 2018; Chu et al., 2019). In human-autonomous vehicle interactions, because high-level vehicles can have social attributes and make moral decisions, the moral type of vehicles may also have an impact on people’s trust in autonomous vehicles (Bonnefon et al., 2016; Martinho et al., 2021). However, there is still a lack of correlation between human–computer trust, especially human-vehicle morality, which is mainly because the moral issues of human-vehicle interaction are relatively complex (Nyholm, 2020). In summary, there is still a lack of systematic and in-depth exploration in the field of human-computer interaction, especially in the field of human-vehicle interaction. Therefore, this study aims to explore the impact of human–vehicle moral matching on trust in autonomous vehicles.

## 2. Literature review and theoretical hypothesis

Trust is a kind of psychological state that refers to positive expectations about the intentions or actions of others (Rousseau et al., 1998). This definition applies not only to human interactions but also to human–computer interactions. However, the difference is that trust in human–computer cooperation is the trust of people in artificial intelligence systems, that is, the human is the trustor, while artificial intelligence systems are the object of trust, that is, the trustee (Nass et al., 1996). However, interpersonal trust is more considered in the context of a binary relationship, with either party as the center, considering the trust of the other party or the trust of the other party (Mayer et al., 1995). Different scholars have different definitions of human-computer trust, and the two more mainstream ones are automation trust and tendency to trust (Lee and See, 2004). This study focuses on people’s trust in trusted objects (autonomous vehicles), not trust tendencies. At present, the research conclusions about human–computer trust are not consistent. Some scholars consider that human–computer trust is similar to interpersonal trust and that the low-level trust generated by human–computer encounters for the first time may increase after direct interaction due to intelligent technology (Muir and Moray, 1996). Some scholars also posit that interpersonal trust can increase through frequent interactions and the passage of time. Human–computer trust is usually not easy to increase but will decline due to technical errors or failures that occur during the interaction (Ray et al., 2008). If the human–computer cooperation task increases the demand and workload too much, the risk of error will also rise. The increase in the cooperation task workload will also lead to a decrease in people’s trust in the artificial intelligence system (Roscoe, 1989). Human operators prefer to perform tasks alone over untrusted automated technology (Chen et al., 2011).

In addition, people’s trust in autonomous vehicles is affected by four factors, including humans, vehicles, the environment and interactions (Chen et al., 2018; Sanders et al., 2019; Sheng et al., 2019). Previous research has focused on people (e.g., their monitoring behavior on vehicles), vehicles (e.g., autonomous driving style, alarm type), and the environment (e.g., driving environment, weather). However, there are few studies on human-vehicle interaction factors. With the emergence of humanoid machines, in complex traffic situations such as manual driving and autonomous vehicles on the road at the same time, we must consider the resulting human–vehicle interaction, which is not only a key factor affecting people’s trust in vehicles but also an important safety guarantee for people to use autonomous vehicles (Raats et al., 2019). In a few related studies, scholars have investigated the merger decision of human–vehicle interaction in time-critical situations (Trende et al., 2019). The results show that when time is tight, participants will use the technological advantages of autonomous vehicles to increase their decision-making time when interacting with highly autonomous vehicles. Thus, the trust in autonomous vehicles by participants with a positive attitude toward technical reliability will increase.

### 2.1. Moral types and trust between human–vehicles

There are various standards to distinguish moral ethics. Among them, deontology and utilitarianism are the most frequently discussed and of the most concern to scholars (Gawronski and Beer, 2017). The deontological core is that the right takes precedence over good and that the observance of moral norms is absolute and nonutilitarian. In contrast, the core of utilitarianism is that human nature is selfish, morality is the means to seek profits, and morality is to obtain utilitarian benefits (Beauchamp and Bowie, 1983; Reidenbach and Robin, 1990). Although some moral-related studies believe that the two are not completely opposed to each other, and that there is a certain relationship between them, people always prefer a certain type of morality and make their own behavioral choices on the basis of specific choices and practices. The trolley problem shows that although people may have other moral types, the decision to kill one or more people is intuitively reflected in the moral types of utility and deontology (Foot, 1967; Greene et al., 2001). However, intelligent machines represented by autonomous vehicles do not have independent moral types for the time being and only show certain moral beliefs according to their behaviors. Relevant studies have pointed out that robots do not have independent morality consistent with human beings; instead, they can only learn the basic morality of human beings and make corresponding ethical decisions based on the basic moral principles of human society, that is, avoid unethical things (Goodall, 2014; Greene, 2016; Nyholm and Smids, 2016).

Morality is the foundation of behavioral decision-making (Kurtines, 1986). In personal choice, people are often selfish; they hope that they can obtain the maximum benefit. Based on considerations of self-interest maximization, people are more willing to become social partners with decision-makers of deontological morality in interpersonal relationships. They think that companions with deontological morality and credibility make more effective use of limited resources at the same time that they take a larger yield for themselves; that is, a deontological moral partner can increase the degree of reliability greater than a utilitarian moral partner (Sacco et al., 2017).

However, in the team’s interpersonal relationships, true personal morality will not be completely displayed in the form of behavioral decisions. On the one hand, because the relationship between both sides is based on them by their behavior, which determines each other’s impression, once their behavior choices are too beneficial for themselves, it will affect their image. On the other hand, as long as the common interests of the team are met, it will not cause dissatisfaction if some of the individual’s interests cannot be satisfied, meaning that individuals will adjust their moral decisions according to the behavioral decisions that result from the moral types of their teammates (Baumard et al., 2013; Bostyn and Roets, 2017); that is, the team has a constraining/limiting effect on the behavior of individuals. Human–computer interaction research generally selects typical moral (utilitarian and deontology) populations for research (Baniasadi et al., 2018a; Liu and Liu, 2021; Yokoi and Nakayachi, 2021). When people think that robots are reliable and trustworthy, human–computer interaction goes more smoothly. When the robot leads incorrectly, it will cause strong distrust among subordinates, and may even lead to decision-making errors and affect the team’s work results. Human-computer trust is particularly important when facing robot leaders, which will be related to the satisfaction of subordinates with the task (Hancock et al., 2011).

The driving behavior of autonomous vehicles influenced by technology is more efficient. In a way, autonomous vehicles will be able to act as their own agents, rather than tools simply controlled by humans, and they will need to act according to some moral and ethical principles. Existing research suggests that autonomous vehicles could have morality, and the type of morality they share with the driver/person will affect the driver/person’s trust in the vehicle. In social interactions, moral types influence the perception of the behavioral expectations of the interaction object. Artificial intelligence products may have social attributes such as morality. In human–autonomous vehicle interactions, a person’s trust in the vehicle is influenced by his or her own moral type. Utilitarian moral individuals are more likely to rely on objects whose behavior is predictable than those of deontological moral types. Besides, studies on human–computer interaction generally select typical moral types (utilitarian and deontology) to conduct research (Baniasadi et al., 2018b; Sivill, 2019; de Melo et al., 2021; Yokoi and Nakayachi, 2021; Nijssen et al., 2022; Vianello et al., 2022). Therefore, our study uses two typical moral types (utilitarian and deontological) to study the impact of the moral types of people and autonomous vehicles on the trust in autonomous vehicles. We propose hypothesis:

*H1*: People’s moral type will affect their trust in autonomous vehicles. Individuals with utilitarian morality have greater trust in autonomous vehicles than individuals with deontological moral.

### 2.2. Perceived value, perceived risk and their mediating role

The emergence of technology not only has value and makes people’s lives more convenient but also has risks because people are not sure whether their expected results are correct. The perceived value and risk of technology is similar to a double-edged sword, which affects people’s use of technology at the same time (Naami et al., 2017).

Perceived value refers to people’s evaluation after weighing the perceived benefits brought by technology and the cost paid (Zeithaml, 1988). At present, studies related to perceived value mainly focus on e-shopping, tourism, services and other fields (Boksberger and Melsen, 2011; Chiu et al., 2014; Haji et al., 2021). It is generally believed that perceived value positively affects people’s trust in related technologies (Bernarto, 2017). In the field of human-vehicle interaction, autonomous vehicles reduce the effort and time investment of drivers in the process of driving, create a convenient travel experience for drivers, and make drivers feel more value beyond manual driving, thus increasing their trust in autonomous driving. A study of the intention to use a shared autonomous vehicle showed that the perceived value of the vehicle has an impact on the intention to use it. When users perceive a higher value, the level of trust in the autonomous vehicles also increases (Häuslschmid et al., 2017).

Perceived risk refers to the possibility of physical, social, and economic damage from the generation of technology, including concerns about uncertainty (the possibility of adverse consequences) and loss (the severity of the consequences; Cox and Rich, 1964; Mayer et al., 1995). Risk is an important antecedent variable affecting trust, which transforms the simple causal relationship between people’s perceived intention and behavior into a more complex conditional relationship (Featherman and Fuller, 2003). People’s perceptions of risk may change due to changes in other conditions, thus affecting the original trust (Veloutsou and Bian, 2008). For example, the inherent pitfalls and low penetration of autonomous vehicles technology may increase people’s perception of its risks, which in turn erodes trust. On the one hand, due to the lack of understanding of autonomous vehicles technology, people have doubts about its functionality and safety (Schwammberger, 2018). On the other hand, the high convenience and comfort of autonomous vehicles may lead to more applications, that is, to an increase in the number of vehicles on the road, which will increase the risk probability (Pek and Althoff, 2019).

Human morality affects human’s perceived value and perceived risk (Gantman and Van Bavel, 2015). The similarities and differences in perceived value could lead to different behaviors. Followers who have the same moral type as leaders in the organization perceive higher value and lower risk, and leads positive results at work (Zhang et al., 2022). In contrast, followers who have different ethical types than leaders feel less value, more risk, and are unable to identify with the organization’s behavior, which go against to team development. In addition, the relationship between morality and perceived value and perceived risk has always been the focus of discussion in the process of technological development which cannot be ignored (Hayenhjelm and Wolff, 2012). The human–vehicle relationship is a good proof that people’s perception of the value and risk of autonomous vehicles is also affected by their own moral level. However, scholars have not reached a consistent answer to this question.

In summary, perceived value and perceived risk are important factors affecting the safety of autonomous vehicles. High trust level is the premise and guarantee of people’s belief in autonomous vehicles safety, but the impact of perceived value and perceived risk on trust and its mechanism on human–vehicle ethics is not clear. Previous research has found that people’s moral type affects their perception of the value and risk provided by the object of interaction (Mortimer et al., 2020). In addition, when people believe that the value of autonomous vehicles is higher, the higher their trust in them, and the higher the risks they pose, the lower their trust in them. Therefore, the following hypotheses are proposed:

*H2*: Perceived value plays a mediating role between people’s moral type and trust in autonomous vehicles.

*H3*: Perceived risk plays a mediating role between people’s moral type and trust in autonomous vehicles.

### 2.3. Moderating effects of moral type of autonomous vehicles

In the human-human team or human–computer team, people’s trust is influenced by their own morality and the morality of the objects they interact with (Friedman, 2020; Malle and Ullman, 2021). The current research on human–vehicle trust mainly focuses on the influence of factors related to people, vehicles and the environment and rarely studies the influence of interaction factors on human–vehicle interactions (Chen et al., 2018; Sanders et al., 2019; Sheng et al., 2019). Although human drivers are less involved in the operation in autonomous vehicles, the use of the autonomous vehicles function mainly depends on their trust in the vehicle (Choi and Ji, 2015). The low participation of humans in autonomous vehicles does not mean that they pay less attention to humans. The beneficiaries of autonomous vehicles are still people, and in this process, the autonomous decision-making and behavior of the vehicles affect people’s trust in the autonomous vehicles. Therefore, interaction factors affect human–vehicle trust.

In the process of human-vehicle interaction, autonomous vehicles do not simply transfer the driving power from the individual to the machine. Instead, it needs to complete the driving task in a way that conforms to social rules; that is, the decisions made by autonomous vehicles reflect their moral choices (Fagnant and Kockelman, 2015). Similar to interpersonal relationships, the human–vehicle relationship needs to consider the impact of their interaction on each other’s decision-making, especially the impact on human–vehicle trust. The influence of this interaction varies by different fields, which is usually present as homogeneity tendency (that is, when the decision-making types of people and vehicles are identical or similar) or heterogeneity tendency (that is, when the decision-making types of people and vehicles are inconsistent or even opposite). In the case of homogeneity matching, for example, in the field of human–computer interaction, the moral and standard behavior of robots leads to the relatively normative and polite behavior of humans (Liu et al., 2022). In group decision-making, people tend to make the same choice as others, and few people stick to their own choice (Herrera-Viedma et al., 2017). In the case of heterogeneous matching, for example, in the experiment with public goods, people appeal to others to make contributions, but they will make a utilitarian choice to hitchhike, reflecting their selfish side (Brown and Sacco, 2019).

In conclusion, autonomous vehicles will play an important role in future society, and people’s trust in them will affect their functions. To a certain extent, autonomous vehicles will be able to act as their own agents, rather than simply tools controlled by humans. That is, they will need to act according to some moral and ethical principles. In addition, moral matching affects perceived value and risk. Specifically, when the vehicle has deontological morality and the person has utilitarian morality, the involvement of the vehicle reminds people not only to consider the expected value but also to pay attention to the principle. At the same time, the deontological moral behavior of the vehicle complements the previous lack of consideration of the principle, so people perceive the vehicle as having greater value and lower risk. Similarly, when the vehicle is utilitarian and the person is deontological, it reminds people that they should not only consider complying with the moral code but should also consider more value on the basis of complying with the moral code and feel that the vehicle is of greater value and lower risk. Therefore, the following hypotheses are proposed:

*H4*: The moral type of the vehicle moderates the relationship between the moral type of the person and the perceived value.

*H5*: The moral type of the vehicle moderates the relationship between the moral type of the person and perceived risk.

**Figure 1:**
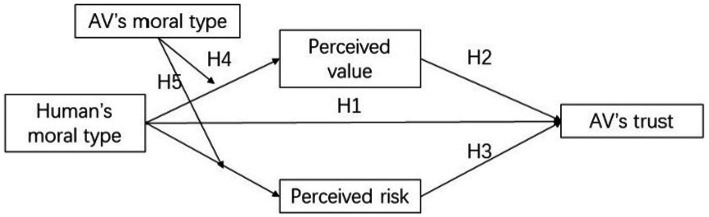
Research framework.

Based on the five hypotheses in this study, we propose a research model framework for the impact of human–vehicle moral matching on trust, which is shown in Figure 1.

## 3. Research method

### 3.1. Measurement

This study included an independent variable, namely, the moral type of the participants (intergroup variable; two levels: deontology and utilitarian); one dependent variable, which is people’s trust in autonomous vehicles; two mediating variables, perceived risk and perceived value; and one moderating variable, the humanized moral type of autonomous vehicles (intergroup variable; two levels: deontology and utilitarian).

The moral types of independent variables are mainly measured by the moral scale compiled by Reidenbach and Robin (1988, 1990). The Cronbach of the original scale is *α* = 0.89. Participants were required to fill in the two dimensions of utilitarian and deontology subscales. The utilitarian scale has a total of nine questions, in order to make the content of items more similar to the context of this study and the participants can better understand the topic. Adjust the original question items according to the actual situation, which is reflected in adjusting the subject of the item from the third person to the first person “I.” For example, “If actions can/cannot be justified by their consequences” is adjusted to “If the behavior of my choice can be confirmed by the results, then I think my choice is fine.” The deontology scale has five questions, and the original subject is also modified to facilitate measurement, representing items such as “Violates an unwritten contract or not” to “I think it is wrong to violate unwritten rules in society.”

The dependent variable trust refers to the trust scale developed by Yokoi and Nakayachi (2019), which has three questions. The Cronbach of the original scale is *α* = 0.84. Although the subject of each question of the original scale is related to artificial intelligence, it is not clear and intuitive enough. To make the item content more relevant and convenient for the participants to understand, the trust object in the topic was clearly defined as automatic driving. For example, the original scale of item “This AC (Mr. A) can be entrusted with driving” is adjusted to “I think the autonomous vehicle is reliable.”

Mediator perceived value uses the perceived value scale compiled by Wang et al. (2004) based on Bourdeau et al. (2002). The Cronbach of the original scale is *α* = 0.87. The scale contains eight questions, such as the auxiliary function “self-driving vehicles satisfy my needs.”

The mediating variable of perceived risk uses the perceived risk scale compiled by Bansal and Kockelman (2016). The original scale contained eight questions. The Cronbach of the original scale is *α* = 0.95. Among the items in the original scale, “I will feel afraid when I use autonomous vehicle technology for the first time” and “I will feel unnecessary nervous when I use autonomous vehicle technology for the first time” have similar significance in this study, so the items are combined to form “I will feel afraid/nervous when I use autonomous vehicles technology for the first time.” Therefore, the scale used in the study contains 7 questions.

**Figure 2:**
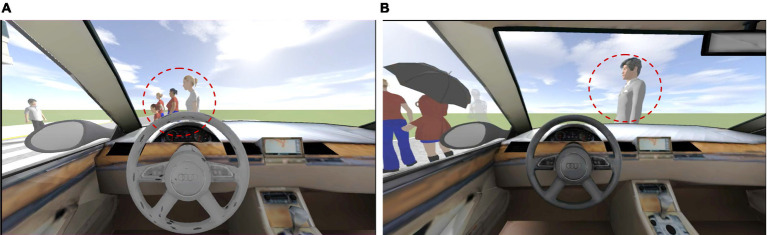
A screenshot of the autonomous vehicle ethical decision video. **(A)** Decision on vehicles of the deontological moral type. **(B)** Decisions on vehicles of the utilitarian moral type. The person in the red dashed circle is the person who is about to be hit during the autonomous driving of the test subject.

The moderating variable, vehicle moral type, was controlled by setting the experimental situation. In different experimental situations (deontological and utilitarian moral type vehicles), participants watch different “trolley problems” videos (Foot, 1967), in which the autonomous vehicles showed different moral decisions. Autonomous vehicle faces driving problems, if the vehicle follows the established route normally, then may hit 5 people. If changes the route, although 5 people are saved, 1 person on the new route will be hit. When the variable of the moral type of the vehicle is the level of deontology, the video scenario is that the vehicle chooses to crash into multiple people in order to save an innocent person. When the moral type variable of the vehicle is utilitarian level, the video scenario is that the vehicle chooses to change route and crash into a person in order to save more lives. The video screenshot is shown in Figure 2. In order to test that participants in the experiment did correctly perceive the moral type of the vehicle in different situations, after the video viewing, the participants are asked to answer two questions to confirm that he was aware of the moral type of the vehicle. The two questions are “Which of the following option is happening in the video you watch?” “In this scenario, do you think the vehicle behaved appropriately?” All participants answered correctly, which means the control variable setting is valid.

In addition, this study contains five control variables: gender, age, year of driving experience, knowledge of autonomous vehicles, and driving experience.

The utilitarianism, deontology, perceived value, perceived risk, and trust scales of this experiment are scored using the Likert five-point scale. The corresponding values of the scale have the following meanings, 1 = strongly disagree, 5 = strongly agree. The gender variable is 0–1. The age and year of driving experience are set by using the single selection method of the interval, such as age: ‘< 20 years old’ = 1, ‘> 60 years old’ = 6; year of driving experience: ‘< 1 year’ = 1,’ > 10 yeas’ = 5. The degree of understanding of autonomous vehicles is based on the Likert five-point scale, 1 = very ignorant, 5 = very knowledgeable. There are two options for driving experience settings: rich and not rich.

### 3.2. Group of subjects

From March 1 to 15, 2022, we released experimental recruitment information through an Internet questionnaire platform in China. To screen out the typical moral type of participants, the participants were asked to fill out the moral scale according to their daily behaviors, including two subscales/dimensions of utilitarian type and deontology type, which introduced in Subsection 3.1. After excluding the answers that did not meet the criteria (e.g., the answer time was too short and all the scores were consistent), a total of 227 potential participants were obtained. According to the scores of the two dimensions, the participants with high utilitarian scores (more than 3.5) and low deontology scores (less than 2.5) and the participants with low utilitarian scores (less than 2.5) and high deontology scores (more than 3.5) were selected as the typical utilitarian and deontological individuals for the experiment. 200 effective typical participants were recruited to participate in the experiment. Among them, 107 were deontological moral participants, and 93 were utilitarian moral participants, which were divided into a deontological group and a utilitarian group.

In the study, to confirm whether the participants had the corresponding typical moral type again, they were asked to answer a binary moral decision question at the beginning of the experiment. The background of the question was in the workplace competition scenario, in which the participants and their best friends participated in the election together; faced with the final binary situation, the participant was aware of a work mistake of his/her friend. If he or she quietly reported the mistake, the friend naturally would lose the opportunity and not know his/her behavior, but the participants succeeded in the election. If he or she hid it silently, the friend would win the election, while the participant would fail. In this case, the participants were asked to choose whether to report it. Reporting is a utilitarian decision while hiding the mistake represents a deontological decision. All utilitarian participants chose to report quietly, while all deontological participants chose to hide it, which means that all participants had the typical deontological/utilitarian moral type.

SPSS software was used to describe the statistical function and analyze the characteristics of the participants. There were 109 female samples (48.02%) and 118 male samples (51.98%). The age group of the sample was mainly from 21 to 40 years old (78.42%). The average age of the samples was 34 years old, minimum = 19 years old and maximum = 63 years old. 178 of them had rich driving experience (66.31%), and 51 of them had not much driving experience (33.69%). On average, participants had a certain level of Familiarity of autonomous vehicles (average understanding = 3.50, SD = 0.92 on a 5-point scoring of Likert). The basic information of the participants is shown in Table 1.

**Table 1:** Statistical characteristics of samples.

Project	Sample distribution	Number of samples	Percentage (%)	Mean	SD	Project	Sample distribution	Number of samples	Percentage (%)	Mean	SD
Age	<20	10	4.41	34.40	.94	Gender	Male	118	51.98	–	–
	21–30	110	48.46				Female	109	48.02		
	31–40	68	29.96			Familiarity of autonomous vehicles	Very ignorant	2	0.88	3.50	0.92
	41–50	31	13.66				Do not know too much	37	16.30		
	51–60	5	2.20				Unclear	53	23.35		
	>60	3	1.32				Understand better	111	48.90		
Year of driving experience	<1 year	23	10.13	2.14	1.42		Very knowledgeable	24	10.57		
	1–3 years	65	28.63			Driving experience	Rich	178	66.31	0.80	0.40
	3–5 years	57	25.11								
	5–10 years	18	7.93				Not rich	51	33.69		
	>10 years	13	5.73								

### 3.3. Experimental design process

This experiment is designed as a 2*2 between-participant design study. In this experiment, the deontology group and the utilitarian group were randomly divided into two groups. These four groups were named Utilitarian Group A, Utilitarian Group B, Deontology Group A and Deontology Group B, respectively. The two Groups A were faced with situations in which the vehicle’s morality was utilitarian (the moral type of the moderating variable vehicle was utilitarian), and the two Groups B were faced with situations in which the vehicle’s morality was deontological (the moral type of the moderating variable vehicle was deontology). The specific grouping is shown in Table 2.

**Table 2:** Groups of participants.

Group	The participants type	Type of video to watch	The number of participants
Utilitarian of group A	utilitarian	utilitarian	51
Utilitarian of group B	utilitarian	deontology	55
Deontology of group A	deontology	utilitarian	38
Deontology of group B	deontology	Deontology	56

The experiment was conducted online, and the experimenter and the participants entered the same online video conference room and turned on the video. Participants were asked to be in a quiet, undisturbed, comfortable environment and to turn on screen sharing. The experimenter guides the participants to complete the experiment through video conferencing software and distributes experimental materials, such as informed consent forms, questionnaires, etc.

Before the start of the experiment, the participants completed the informed consent form, were informed of the experimental content and answered the ethical decision-making question introduced in Subsection 3.2 paragraph 2. After the start of the experiment, all the participants watched an introduction video of autonomous vehicles to preliminarily understand autonomous vehicles. The video content included the driving situation and the development of automatic vehicles in common situations, such as high-speed sections, urban roads and crossings. Its duration was 130 s. Participants were told to take a one-minute break. Second, according to the group, the participants watched the “trolley problem” video of deontology or utilitarian vehicles, and the duration of the two videos was 73 s. Then participants needed to answer two questions about vehicle moral type perception (introduced in Subsection 3.1 paragraph 6). Through their respective videos, participants learned about the consequences of the choice of autonomous vehicles in their participating situations—killing one or five people. Finally, the participants were asked to complete the experimental questionnaire, including the moral perception of the behavior decision of the autonomous vehicle in the video, perceived value and perceived risk, trust in autonomous vehicles, and demographic information. The experiment took about 10 min.

## 4. Data analysis and results

SPSS software was used for data analysis in this study. It consists of two steps. First, the descriptive statistics of variables are performed by the descriptive statistical function under the analysis, and the manipulation check is performed by the independent sample *t*-test and correlation test under the analysis. Second, the multiple linear regression method is used to examine the influence of the moral type of the independent variable on the trust of the dependent variable layer by layer. The mediating role of perceived risk and perceived value between human moral types and trust; and examining the moderating effect of the moral type of the vehicle on the relationship between human moral type and trust.

### 4.1. Manipulation check

The results of the reliability test showed Cronbach’s alpha values of each variable. The Cronbach’s alpha for utilitarian was 0.707, the Cronbach’s alpha for deontology was 0.924, the Cronbach’s alpha for perceived value was 0.887; the Cronbach’s alpha for perceived risk was 0.900; the Cronbach’s alpha for trust was 0.838, which were all higher than standard 0.7. Besides, we added the KMO test and the Bartlett spherical test to this basis, in which the KMOs of utilitarianism, deontology, perceived risk, perceived value and trust are 0.697, 0.922, 0.918, 0.880, 0.713, which are all greater than 0.6. In addition, the significance of the Bartlett spherical test is < 0.001, indicating that the variables used in this study have high reliability. Therefore, the statistics showed that the reliability of these five scales was acceptable.

According to the results of the independent sample ANOVA test, there were no significant differences in gender, age, driving experience, driving age or knowledge of autonomous vehicles among the four groups (all *p* > 0.05), as shown in Table 3. These results indicate that further data analysis can be carried out.

**Table 3:** Results of ANOVA-test comparing between two groups of participants ANOVA.

Variable	The utility of group A		Deontology to group A		The utility group B		The deontology of group B		*T (198)*	*p*
	Mean	SD	Mean	SD	Mean	SD	Mean	SD		
Gender	–	–	–	–	–	–	–	–	0.579	0.887
Age	30.2	1.03	36.8	1.07	32.6	0.98	37.8	0.99	2.008	0.135
Driving years	2.04	1.37	2.06	1.57	2.26	1.27	2.21	1.45	0.497	0.685
Driving experience	3.80	0.40	2.76	0.43	3.87	0.34	3.80	0.40	1.232	0.685
Autopilot knowledge level	3.60	0.92	3.37	0.99	3.68	0.87	3.41	0.87	1.232	0.299
Perceived value	3.24	1.02	3.05	1.02	3.73	0.85	2.98	0.93	–	–
Perceived risk	1.71	0.95	3.25	1.02	1.62	0.88	2.22	1.02	–	–
Trust	3.36	0.96	3.08	0.99	3.80	0.85	2.99	1.00	–	–

According to the results of the correlation test, there is a significant correlation among utilitarianism, deontology, perceived risk, perceived value and trust (all *p* < 0.05), as shown in Table 4.

**Table 4:** Variables mean, standard deviation, correlation coefficient and internal consistency of the scale.

	*M*	SD	Utilitarian morality	Obligatory morality	Perceived value	Perceived risk	Trust
Utilitarianism	3.34	0.96	0.707^#^				
Deontology	2.96	0.74	0.418^**^	0.924^#^			
Perceived value	3.28	0.98	0.183^**^	0.579^**^	0.900^#^		
Perceived risk	2.79	0.92	−0.240^**^	−0.557^**^	−0.544^**^	0.887^#^	
Trust	3.08	0.94	0.178^*^	0.486^**^	0.744^**^	−0.457^**^	0.838^#^

^*^indicates significance at 0.05 level, ^**^indicates significance at 0.01 level, and ^#^indicates Cronbach’s *α* value.

The above results indicate that the following data test can be carried out.

### 4.2. Hypothesis testing

In this study, the multiple linear regression method of least squares estimation was used to test the hypothesis. Model 1 only considered the relationship between independent variables (human moral type) and dependent variables (trust), the influence of the moral type of the independent variable on the human-vehicle trust of the dependent variable, and did not introduce the mediating variable and the regulating variable into the model. The results of regression analysis showed that the moral type of the person had a significant impact on the trust degree of autonomous vehicles (Model 1, *R*^2 = 0.086, *F*(8,191) = 3.034; The type of morality of the person, *β* = 0.529, *p* < 0.001), that is, assuming that H1 is verified, the more utilitarian the person, the higher the trust in the autonomous vehicles.

Model 2 considered the relationship between the independent variables and the mediating variables of perceived value and perceived risk. The results showed that the regression effect of human morality on perceived value and perceived risk was significant (Model 2, *R*^2 = 0.844, *F*(8,191) = 129.277; perceived value: *β* = 0.753, *p* < 0.001; perceived risk: *β* = −0.219, *p* < 0.001). Considering the relationship between human morality, perceived value, perceived risk and trust, the results showed that the regression effect of perceived value on trust was significant (Model 3A: *R*^2 = 0.068, *F*(8,191) = 2.353; perceived value: *β* = 0.460, *p* < 0.01; Model 3B: *R*^2 = 0.063, *F*(8,191) = 2.176; perceived risk: *β* = −0.403, *p* < 0.01), while the regression effect of human morality on trust was not significant (Model 2: *β* = 0.117, *p* > 0.05). In other words, Hypotheses H2 and H3 were verified, and perceived value and perceived risk played a fully mediating role in the impact of human morality on trust in autonomous vehicles.

**Figure 3:**
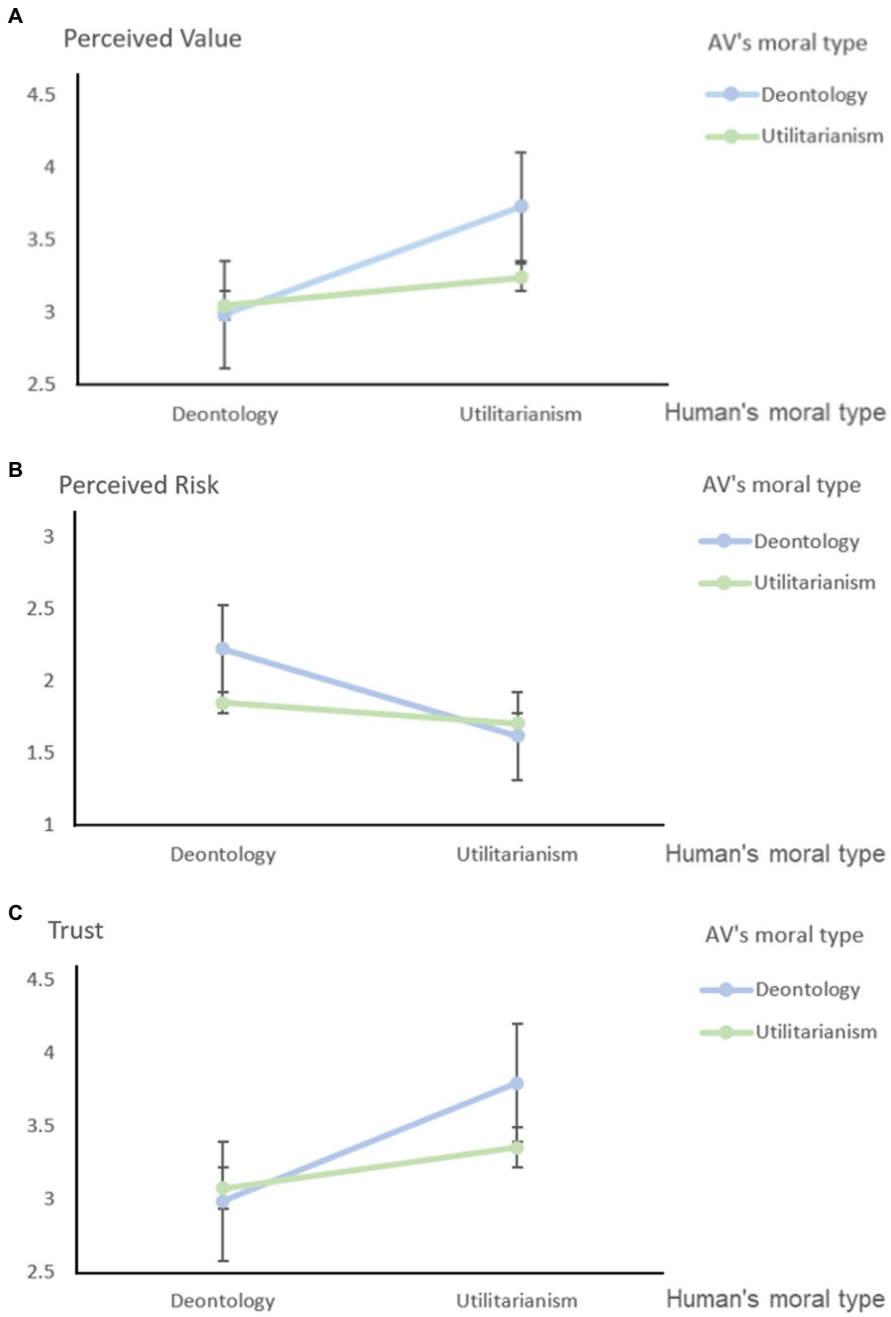
**(A)** The moderating effect of vehicle moral type on the impact of human moral type on perceived value. **(B)** The moderating effect of vehicle moral type on the impact of human moral type on perceived risk. **(C)** Trust change diagram of the similarities and differences of moral types of people and vehicles.

Considering the role of moderating variables, the results showed that the interaction between vehicle morality and human morality had significant effects on perceived value and perceived risk (Model 4A: *R*^2 = 0.088, *F*(8,191) = 2.299; *β* = 0.137, *p* < 0.05; Model 4B: *R*^2 = 0.096, *F*(8,191) = 2.534; *β* = −0.140, *p* < 0.05), which affects people’s perceptions of the value and risk of autonomous vehicles. In other words, vehicle morality plays a partial moderating role in the research on people’s moral trust in autonomous vehicles, which means that Hypothesis H4 is verified. Under the control of the vehicle’s moral type variable, when the vehicle was deontology (0), the impact of the person’s moral type on the perceived risk and perceived value was significant (both *p* < 0.01). When the vehicle is utilitarian (1), the moral type of the person has no significant effect on perceived risk or perceived value (both *p* > 0.1). Furthermore, when the vehicle is utilitarian and the person is utilitarian, people’s perceived risk, perceived value and trust in the vehicle are higher than those in the other three experimental situations. The details are shown in Table 5 and Figure 3.

**Table 5:** Results of linear regression analysis on acceptability.

Variable types	Variables’ name	Model 1	Model 2	Model 3		Model 4	
		Trust	Trust	Perceived value 3A	Perceived risk 3B	Perceived Value 4A	Perceived Risk 4B
Independent variables	Human’s moral type	0.529^***^	0.117	0.460^**^	−0.403^**^	0.390^**^	0.485^**^
Mediating variable	Perceived value		0.753^***^				
	Perceived risk		−0.219^***^				
Moderator variable	The moral type of the vehicle					−0.152	−0.182
	Human moral type^*^ vehicle moral type					0.137^*^	−0.140^*^
Control variables	Age	0.092	0.034	0.079	0.004	0.005	0.069
	Knowledge of autopilot	0.085	0.082	0.042	0.132	0.134	0.037
	Gender	0.043	−0.011	0.110	0.129	0.139	0.107
	Driving experience	−0.284	−0.029	−0.311	0.091	0.078	−0.322
	Driving years	0.057	−0.003	0.090	0.034	0.035	0.089
	*R* square	0.086	0.844	0.068	0.063	0.088	0.096
	The adjusted *R* square	0.058	0.838	0.039	0.034	0.050	0.058
	*F* (8,191)	3.034	129.277	2.353	2.176	2.299	2.534

^*^indicates significant association at the 0.05 level (two-sided), ^**^indicates significant association at the 0.01 level (two-sided), ^***^indicates significant association at the 0.001 level (two-sided).

In general, all hypotheses involved in the research model of the impact of human-vehicle moral matching on trust are verified. In addition, according to the confirmatory factor analysis results, Chi/DF = 2.557 (less than the standard value of 3.000), GFI = 0.935 (more than the standard value of.900), RMSEA = 0.089 (less than the standard value of.900), indicating that the model has a good fit.

## 5. Discussion

Hypothesis 1 of this study is verified. Individual moral type affects the degree of trust in autonomous vehicles. Utilitarian moral individuals have greater trust in autonomous vehicles than deontological moral individuals, which is consistent with previous research conclusions on the relationship between moral type and trust (Bonnefon et al., 2015; Li et al., 2016; Malle et al., 2016). Without considering the moral type of vehicles, as the only moral subject, people’s trust in autonomous vehicles mainly depends on their dependence on the value of them. Utilitarian moral individuals are more likely to rely on objects whose behavior can be expected, such as more efficient autonomous vehicles. In addition, utilitarian moral individuals are more sensitive to the innovativeness and functionality of new technologies/products, so they perceive a higher level of dependability and trust in autonomous vehicles than other moral types (Liu and Liu, 2021).

Hypotheses 2 and 3 of this study have been verified. Both perceived value and perceived risk play a mediating role in the influence of human moral type on the trust of autonomous vehicles. Human moral type affects their perception of the risk and value of autonomous vehicles, while perceived risk negatively affects and perceived value positively affects people’s trust in autonomous driving technology, that is, perceived value and perceived risk present a double-edged sword effect, and people are more willing to avoid risks and obtain benefits at the same time (Chen et al., 2022). Similar to other emerging technologies, on the one hand, the innovation of autonomous vehicles can enhance people’s perception of realism and value of driverless technology. On the other hand, it will also increase people’s awareness of risks. The former will have a positive impact on technology evaluation, increase trust and technology acceptance, and promote purchase behavior. The latter has a negative impact on technology, leading to a decrease in trust and purchase intention. Due to these conflicting effects, autonomous vehicle manufacturers and researchers face the challenge of recognizing the role of perceived value and risk in the development of technology, and balancing risks and benefits so that users can both avoid risks and gain more benefits when using new technologies.

Hypotheses 4 and 5 of this study are verified. The moral type of the vehicle plays a moderating role in the trust model of human morality on autonomous vehicles, specifically adjusting the influence of human’s moral type on perceived value and perceived risk. This is in line with the previous studies that showed the human’s moral type influence on trust is affected by other factors (Waytz et al., 2014). These include the moral type of the vehicle, that is, the human–vehicle moral matching has an impact on trust (mediated by perceived risk and perceived value).

Furthermore, the results of this study suggest that heterogeneous person-vehicle moral matching (i.e., people are utilitarian, and vehicles are deontology) has a more positive effect on trust than homogeneous moral matching (i.e., both vehicles and people are deontology or both are utilitarian). It is inconsistent with previous studies. Based on the theory of shared values, some scholars proposed that when people and their agents have homogenous values, their trust in the agent will increase (Earle and Cvetkovich, 1997; Bhardwaj and Sergeeva, 2023); that is, when people realize that their value of things is consistent with their agent values the same things, they will trust the agent. However, the conclusion of this study is not completely consistent with the theory of shared values, which may need to be explained by the hypothesis of individual selfish preferences (Gill, 2020). This is consistent with the previous conclusion of Shariff et al. (2017), who believed that autonomous vehicles should operate under utilitarian principles, but human preferred to buy vehicles that prioritized their own lives as passengers.

The individual selfish preference hypothesis holds that people are selfish and their behavior seeks to maximize their own interests. With utilitarian type moral individuals hopes that their own has a deontological partner in a team, because the partner will be more dedicated to the collective. In this case, individual does not need to pay much to gain their profits, but this is only from the perspective of individual selfish decision-making, which may lead to the failure of collective decision-making. It can also cause problems in community relations and brings an imbalance between collective interests and individual relations. This means that individuals may make choices at the expense of others’ interests to maximize their own interests when making decisions. The common “free-riding” phenomenon and “public goods experiment” in life are typical examples.

It is worth noting that the combination of deontological human and utilitarian vehicles has greater trust than the combination of utilitarian human and utilitarian vehicles. It shows that the selfish tendencies of individuals play a more important role in the moral decision-making of human–car interaction. This is consistent with previous research that deontological moral peers in teams are more likely to cultivate the perception of trust than peers with utilitarian morals (Sacco et al., 2017), which means people in a team tend to be selfish (utilitarian) but expect others to be deontological and make ethical appropriate contributions. This has also been demonstrated in (Everett et al., 2016), where people take advantage of the law-abiding and prudent driving strategies of the autonomous vehicles and make decisions in their favor on this basis. They also consider that decision-makers are obligated to be more moral and trustworthy. Our analysis suggests that pure selfishness, which underlies human motivation, should probably be replaced by a mixture of selfishness and morality. Besides, human–vehicle moral matching can help increase trust, and the vehicle’s moral behavior needs to be adjusted to the person’s behavior. This matching can improve the person’s trust in autonomous vehicle technology. Based on our findings, the application of successful ethics, which is set by default by most automobile manufacturers to the moral code of autonomous vehicles, deserves further discussion (Faulhaber et al., 2019). In the future, with the deepening of research, the design of autonomous vehicles will need to be adjusted. In addition to focusing on autonomous vehicles technology, it is also necessary to deeply consider the social attributes of autonomous vehicles with increasing artificial intelligence. For example, autonomous vehicles could have certain moral attributes in human society, and this moral attribute will affect their autonomous vehicles decisions in special traffic situations. In addition, for different users, the moral attributes of autonomous vehicles should be able to change with the differences in the moral attributes of different users, providing a way to replace the original single moral system, in order to fully capture the flexibility of human moral judgment, rather than an immutable fixed system, so as to match the moral attributes of people and vehicles, which can enhance the trust of future car users in autonomous vehicles, which is of great significance to traffic safety and security. This may be more helpful for the large-scale realization of autonomous vehicles.

There are still several limitations in this study. First, we selected only two types of typical morality (deontology and utility). In addition, there are other types of morality, and some individual moral types are not typical. Their trust in autonomous vehicles and its mechanism of are worth further exploration based on our study. Second, this study draws on the mature experimental paradigm of the “trolley problem” to measure moral types and abstracts the complex human-vehicle interaction situation into a simple experimental task. In future research, it is necessary to consider longer experimental scenarios and more complex tasks of human–vehicle interaction to provide more empirical results from related studies in the field of human–vehicle interaction trust and autonomous vehicles. This research mainly focuses on human-vehicle moral matching. Although “trolley problem” is mature and classic, with more in-depth research in the field of morality, scholars also point out the deficiency of the trolley dilemma. In future studies, scholars can consider more complicated ethical issues to explore human–vehicle matching moral influence on autonomous vehicles trust, and the differences between interpersonal trust and human–computer trust in the field of autonomous vehicles. Some of the participants have shorter driving experience, and their cognition of driving and autonomous vehicles may be different from that of people with longer driving experience, so this study introduces driving age as a control variable into data analysis to control its impact on the results of the study. Future research can carry out more in-depth human-vehicle interaction research for people with different driving ages and driving experience. Finally, this study explores the impact of human–vehicle moral matching on trust in autonomous vehicles, which can be extended to other in-depth moral issues, including human moral beliefs, to explore trust in the context of human–computer interactions in the future.

## 6. Conclusion

Ethical issue is an important factor affecting trust in human-vehicle interactions and also a key feature of the future development of autonomous vehicles. From the perspective of human–vehicle moral matching, this study explores the influence of human moral type on trust in autonomous vehicles and studies the mediating effect of perceived risk and perceived value, as well as the moderating effect of vehicle moral type. In this study, the classic trolley problem paradigm was used to design videos and set the moral type of autonomous vehicles. A total of 200 participants were recruited to complete the experiment. The results show that people’s typical moral types affect their trust in autonomous vehicles, and utilitarian individuals have greater trust in autonomous vehicles than deontological individuals, which is consistent with the research conclusions related to interpersonal trust. Second, perceived value and perceived risk have a double-edged sword effect; that is, people’s moral type has a positive impact on trust through perceived value and a negative impact through perceived risk, which means that people seek to obtain value and avoid risk as much as possible. Finally, the moral type of the vehicle plays a moderating role in the model; that is, human–vehicle moral matching influences trust through perceived risk and perceived value. Furthermore, heterogeneous moral matching (people are utilitarian, vehicles are deontology) has a more positive effect on trust than homogenous moral matching (people and vehicles are both deontology or utilitarian), which is consistent with the hypothesis of individual selfish preferences. The conclusions of this study provide theoretical extensions to the fields related to human–vehicle interactions and the social attributes of AI and provide exploratory suggestions for the functional design of autonomous vehicles. This conclusion provides a theoretical extension for the fields related to human–vehicle interaction and the social attributes of AI. Further study could explore the influence of moral types, in particular, the impact of human–vehicle moral matching on human-vehicle trust, and reveal the influence mechanism. In addition, exploratory suggestions are made on the social attributes of highly intelligent autonomous vehicles, which can have certain moral attributes in the future to support driving decisions in special traffic situations. Autonomous vehicles could bring fundamental changes to future lifestyles, and some forward-thinking about ethical design and policy can help guide the development of the technology before the technology pitfalls emerge. Autonomous vehicles may bring about fundamental changes in future lifestyles, and some forward-looking thinking on ethical design and policy can help guide the development of technology before technological pitfalls emerge.

## Data availability statement

The original contributions presented in the study are included in the article/Supplementary material, further inquiries can be directed to the corresponding author.

## Ethics statement

The studies involving human participants were reviewed and approved by the Beijing University of Chemical Technology, China. The participants provided their written informed consent to participate in this study. This study was conducted in accordance with the Declaration of Helsinki. The patients/participants provided their written informed consent to participate in this study.

## Author contributions

NC performed the experiment. YZ and NC contributed significantly to analysis and manuscript preparation. YZ performed the data analyses and wrote the manuscript. JS helped perform the analysis with constructive discussions. All authors contributed to the article and approved the submitted version.

## Funding

This article received support from the National Natural Science Foundation of China 72201023, the Ministry of Education of Humanities and Social Science project 19YJC840002.

## Conflict of interest

The authors declare that the research was conducted in the absence of any commercial or financial relationships that could be construed as a potential conflict of interest.

## Publisher’s note

All claims expressed in this article are solely those of the authors and do not necessarily represent those of their affiliated organizations, or those of the publisher, the editors and the reviewers. Any product that may be evaluated in this article, or claim that may be made by its manufacturer, is not guaranteed or endorsed by the publisher.
